# Health utilities in cancer patients: A study protocol for a prospective, longitudinal cohort using online survey

**DOI:** 10.1097/MD.0000000000014647

**Published:** 2019-03-01

**Authors:** Thomas G. Poder, Nathalie Carrier, Nathalie McFadden, Michel Pavic

**Affiliations:** a UETMISSS and CRCHUS, CIUSSS de l’Estrie—CHUS, 1036 Belvedere Sud, Hôpital Youville; b CRCHUS, CIUSSS de l’Estrie—CHUS; c Département de chirurgie, Université de Sherbrooke; d Département de médecine, Université de Sherbrooke, Sherbrooke, QC, Canada.

**Keywords:** breast cancer, colorectal cancer, discrete choice experiment, health preference, health utilities, quality-adjusted life year, time-trade-off

## Abstract

**Background:**

Cost-utility analysis (CUA) is becoming more commonly used in healthcare decision-making. CUA uses the quality-adjusted life-years (QALY) metric, which combines the length of life with the health-related quality of life (HRQoL). Most QALY-measuring instruments were validated for general populations. For patients with cancer, the perception of their health state is different and may vary by the type of cancer considered. In Quebec, no preference weights for QALY have been developed, neither for the general population nor particular subpopulations.

**Methods/design:**

This survey is a prospective, longitudinal cohort study. The study objectives are: to assess the extent of difference in health utilities between the general population and patients with breast or colorectal cancer; to develop a QALY preference weights dataset for patients with cancer; and to perform “mapping” with different HRQoL questionnaires by correlating the SF-6Dv2 with the EQ-5D-5L, European Organization for Research and Treatment of Cancer Quality of Life Questionnaire C30, and functional assessment of cancer therapy - general questionnaires. Data will be collected via a self-administered online survey. Patients’ health utilities will be measured within 2 days before the beginning of a chemotherapy treatment cycle and about 8 days after the start of the chemotherapy. Health utilities will be measured by a hybrid method using the time-trade-off and discrete choice experiment methods.

**Ethics and dissemination:**

The proposed research was reviewed and approved by the Institutional Research Ethics Review Boards of the CHUS. We will disseminate our study findings through peer-reviewed publications and conference presentations.

## 1 Introduction

The health outcomes of different interventions are expressed in terms of their impacts on the quality and quantity of life.^[1]^ Many psychometric instruments are available to measure health-related quality of life (HRQoL). Some of these instruments are generic (eg, the short form 36 (SF-36), Sickness Impact Profile, and Nottingham Health Profile), whereas others were specifically developed for cancer populations (eg, the European Organization for Research and Treatment of Cancer Quality of Life Questionnaire C30 (EORTC QLQ-C30), functional assessment of cancer therapy - general (FACT-G), and functional living index - cancer). These instruments are validated and correlate fairly well with clinical indicators of changes in health status^[2–8]^; however, these instruments do not take into account the relative preferences of individuals for the different dimensions of health that compose these instruments (ie, the weight given to these dimensions does not take into account the patient's preferences). For this reason, these instruments are not appropriate for performing cost-effectiveness analyses that compare the effects of different interventions.^[9]^

Two different interventions for the same health problem can lead to different effects on the various dimensions of HRQoL (eg, pain, anxiety, or functional capacity).^[10,11]^ Without knowing the individual's preferences for these different dimensions, it is very difficult to determine precisely which treatment really improves the patient's HRQoL.^[9]^ Generic or specific instruments, such as the SF-36 or FACT-G, consider these different dimensions as equivalent,^[5,12]^ but this is not the case^[13–15]^; therefore, the continued use of these HRQoL measures in cost-effectiveness studies biases the results in favor of certain interventions that affect health dimensions that are over or underestimated in their conversion formula (ie, the mathematical formula that is used to convert the answers given to a questionnaire into a HRQoL score).

In addition, when one intervention is more efficient and costly than another, a health decision-maker wants to know how much benefit he can expect for each monetary unit invested and have a measure of the benefit that can be compared with other health interventions for different health problems. One way to achieve such an appropriate and scientifically valid comparison is to use instruments that take into account individuals’ preferences for different health states and standardize them into a common unit of measure.^[16]^ These preferences can be assessed by comparing different health states and asking individuals to make choices.^[17]^ It is within this framework that the concept of the cost-utility analysis (CUA) has been put in place.^[18,19]^ CUA is said to be utilitarian because it seeks to measure the utility that individuals derive from a particular situation, in this case the utility or the satisfaction of living in a given health state. To measure the health effects of an intervention, the CUA uses quality-adjusted life-years (QALY), which combines the quantity of life with the HRQoL into a single score.^[18,20]^ This combination makes it possible to compare different treatments and health problems in terms of the cost per QALY gained as a result of the interventions made. However, no QALY instrument has been developed for cancer patients, particularly non-naive patients, because the QALY was originally developed to measure the health utilities of the general population.^[21]^ It is, however, unlikely that cancer patients place the same weights on the different dimensions of health as the general population.^[22–26]^

### 1.1 Study objectives

The objectives of this project are as follows:

1) to assess the extent of difference in health utilities between the general population and patients with breast or colorectal cancer in Quebec;
2) to develop a preference weights dataset for patients with cancer;
3) to perform “mapping” with different HRQoL questionnaires by correlating the SF-6Dv2 with the EQ-5D-5L, EORTC QLQ-C30, and FACT-G questionnaires.

### 1.2 QALY instrument and extant literature

In this study, we will use the latest version of the SF-6D. This questionnaire has been developed by Brazier et al,^[27–29]^ and is derived from the SF-36v2. The SF-36v2 is frequently used in clinical research, and therefore our results will be easily exportable. To date, few adaptations have been made to take into account differences in health preferences between populations. In a recent systematic review, we identified 21 articles that used the SF-6D to develop preference weights in different countries.^[30]^ These articles are based on 9 databases collected in 8 different countries (ie, the United Kingdom [UK], Spain, Portugal, Brazil, the United States, Hong Kong [2], Japan, and Australia). All of these studies were based on the general population. While the study conducted in Portugal^[31]^ indicates few differences from the UK weighting system, those conducted in Hong Kong^[32]^ and Spain^[33]^ show marked differences; these results suggest the existence of cultural differences in health utilities across countries.

To our knowledge, while QALY instruments have been validated for general populations, this has not been the case for subgroups of those populations, which have very different preferences.^[22–26]^ Indeed, it is unlikely that patients will give the same weights to the different dimensions of health as the general population.^[22–26]^ This situation is particularly problematic for populations with cancer, because Garau et al^[24]^ indicate that when the QALY is calculated based on the preferences of the general population, the results are relatively insensitive to health state changes, indicating that using the general population's preferences to calculate QALYs may be inappropriate because they cannot understand what it really means to live with cancer. In addition, Holzner et al^[34]^ have shown that, within groups of patients with cancer, the patient's perception of their health state varies according to the type of cancer considered.

In Quebec, no QALY instrument (SF-6D or other) has been adapted to its linguistic and sociocultural context, neither for its general population nor any particular subpopulation; it is therefore important to develop a preference weights dataset specifically for Quebec. Developing such a dataset will allow researchers to use this utility instrument in a scientifically valid way and allow physicians and decision-makers to choose between different possible interventions based on the real preferences of the individuals concerned.

## 2 Methods

### 2.1 Study design

This survey is a prospective, longitudinal cohort study. Patient outcomes will be measured twice: within 48 hours before the beginning of the chemotherapy treatment cycle and about 8 days after the start of the chemotherapy. The survey of the general population will be done through an online survey company (random selection), and the data will come from another research project already approved by our institution. The methodology of this part of the survey will not be detailed here, but subjects will respond to the same questionnaires (without the specific modules on cancer), which will allow us to compare the general population's survey responses to those of patients with cancer.

### 2.2 Study population

The target population consists of patients with breast cancer or colorectal cancer. These cancer categories were selected based on the number of new cases per year in Quebec and the potential impact of the type of treatment on the HRQoL; treatments for these cancers are particularly toxic.^[5]^ The inclusion criteria are that the patients are 18 years of age or older; reside in Quebec; have already had a chemotherapy treatment cycle^[34]^; be on the eve of a new round of chemotherapy treatment^[5,6]^; and have breast or colorectal cancer. The exclusion criteria are that the patients are over 80 years of age^[6]^; are unable to complete an online questionnaire; are unable to read or write in French; are unable to sign a consent form; the only treatment offered is surgery; the presence of metastases to the brain^[5]^; or have delirium, psychosis, or severe depression.^[5]^

### 2.3 Patient's recruitment

The list of patients’ appointments in chemotherapy clinics is available in a computerized system 1 to 3 weeks before their appointment. Prescreening data will allow us to determine each potentially eligible patient. These patients will first be approached by the referring physicians, who will introduce the study and ask them if they agree to be contacted by a research assistant. Among the patients who agreed to be solicited, selections will be made based on the inclusion and exclusion criteria. All eligible patients will be asked to participate. An appointment will be made with the patient to formally explain the project and sign the consent form. After signing the consent form, the patient will complete the first questionnaire of the study.

### 2.4 Data collection

The data will be collected via a self-administered online survey. Each questionnaire has 4 main components: a sociodemographic component, a section with a set of health states to evaluate using choice tasks, a component to determine the level of difficulty experienced during the completion of the questionnaire, and a component with various HRQoL questionnaires. The survey will require about 40 to 60 minutes to complete. The same questionnaire, without the sociodemographic component, will be used in the second round of the survey (ie, 8 days later).

The first questionnaire will be completed on site with a laptop in which the survey will be accessible via an online platform. Each patient will have a personalized code. Completion of the survey in the second round will be done at the patient's home or at the clinic if he/she is hospitalized or does not have access to a laptop. In each case, a phone call will be made the day before by a research assistant to remind the patient to complete the survey.

Medical data will be collected from patients’ medical records; this authorization will be obtained at the time of signing the consent form.

### 2.5 Research measures

#### 2.5.1 Sociodemographic and medical variables

The sociodemographic questionnaire will collect various data, including age, sex, weight, height, marital status, occupation, education, annual income, health history, and various attitudinal variables. Questions are validated for their univocity by people external to the research team. With the exception of a few statements requiring open responses, most statements will require closed-choice responses.

The main medical variables collected from the patient records will be: cancer site; histopathological classification; stage of progress at diagnosis (I–IV); modality and treatment characteristics (drug, dose, frequency, current cycle); number of treatments already performed; localized or metastatic cancer; adjuvant or palliative; and number of months since diagnosis.

#### 2.5.2 HRQoL questionnaires

Of the HRQoL questionnaires, 2 are generic (SF-6Dv2 and EQ-5D-5L) and 2 are cancer-specific (EORTC QLQ-C30 and FACT-G). Each specific questionnaire is associated with the module corresponding to the cancer site.^[35,36]^ The SF-6Dv2 questionnaire uses a standardized health state descriptive system consisting of 6 dimensions. These 6 dimensions are physical functioning, role limitation, social functioning, pain, mental health, and vitality. All the dimensions, except for pain, have 5 levels; pain has 6 levels.^[37,38]^ Different from the SF-6Dv2, the EQ-5D-5L questionnaire descriptive system consists of 5 dimensions (mobility, self-care, usual activities, pain/discomfort, and anxiety/depression) with 5 levels of severity. All the HRQoL questionnaires used in this study are validated in French Canadian. While the SF-6Dv2 and EQ-5D-5L allow the calculation of QALYs, the EORTC QLQ-C30 and FACT-G only provide HRQoL scores.

#### 2.5.3 Health preferences

**Figure 1:**
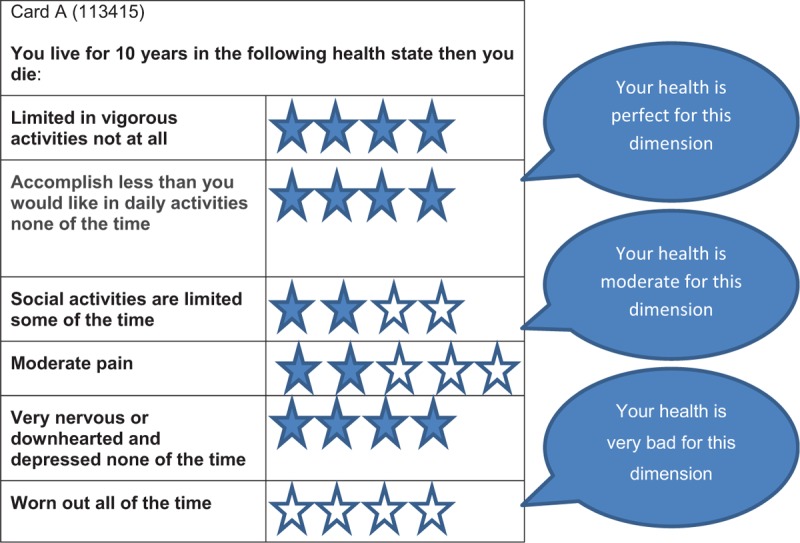
Health state card. This is an example of health state card. There will be 3 intermediate health state cards—a perfect health state card (111111), a worst health state card (555655), and a card for immediate death. The respondent will rank them from 1 to 6, where 1 is the best health state condition and 6 is the worst health state condition or death.

Patients will be first introduced to the notion of a choice task by ranking different health states from 1 to 6, where 1 is the best health condition and 6 the worst health condition or death. The health state conditions were created from the 6 dimensions of the SF-6Dv2. To reduce the cognitive effort that respondents will have to provide to perform their choice task and better identify the different dimensions of health in the SF-6Dv2, a system of symbols will be used. Figure 1 presents an example of a health state card with the interpretation of dimensions and symbols. Patients will then be randomly assigned to a choice set of health states to elicit their health preferences with a hybrid method using the time-trade-off (TTO) and discrete choice experiment (DCE) methods.^[39]^

#### 2.5.4 The TTO method

**Figure 2:**
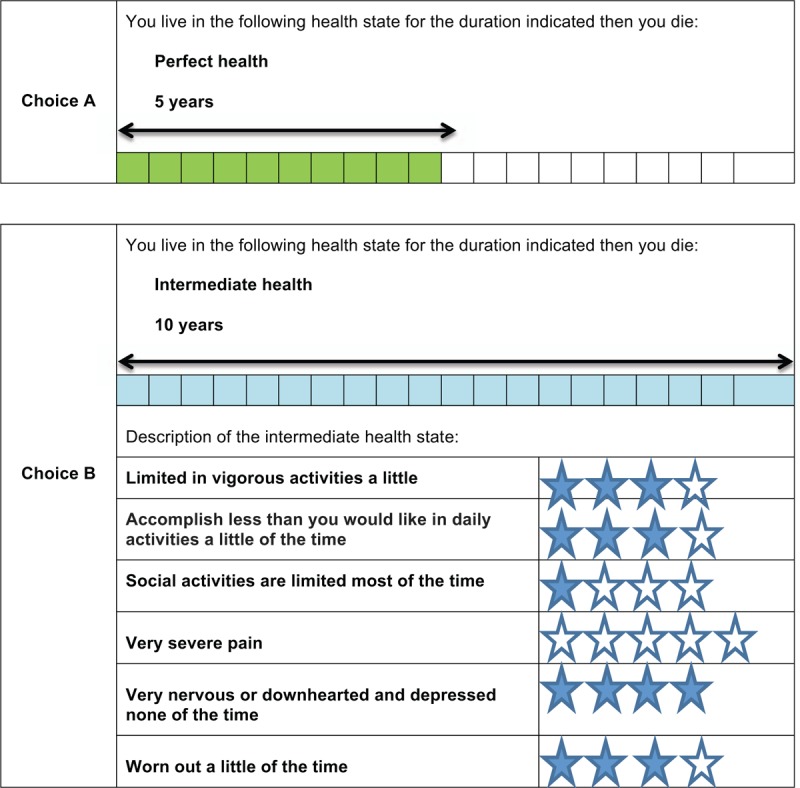
Health state card using the time-trade-off (TTO) choice method. Respondents have to make a choice between 2 health states (ie, a perfect health state [choice A] and an intermediate health state [choice B]) by varying the life span in the perfect health state.

**Figure 3:**
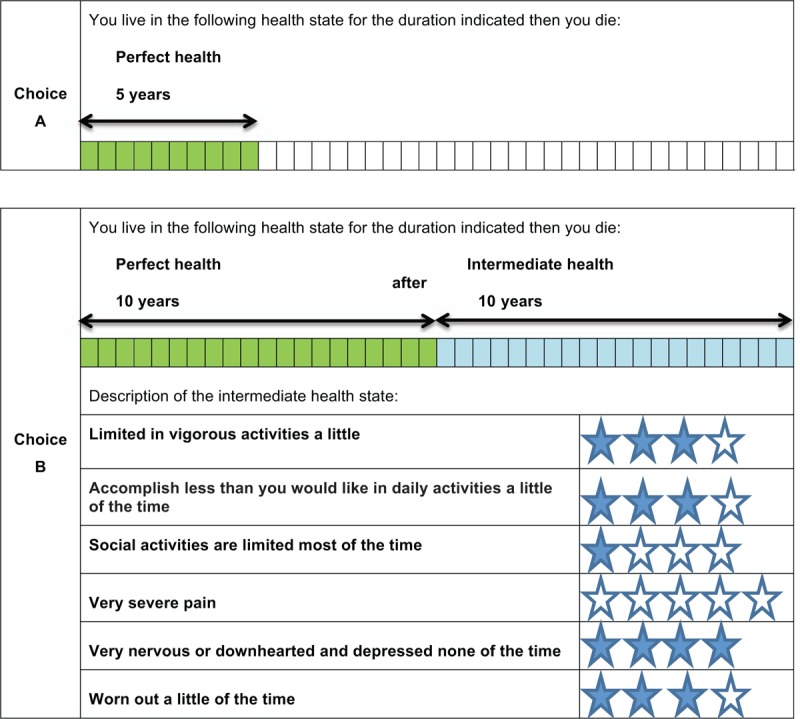
Health state card using the time-trade-off (TTO) choice method. Choice B is a combination of the perfect health state and the intermediate health state.

The version of the TTO used in this study is the EuroQol Valuation Technology (EQ-VT).^[40]^ The TTO method evaluates the length of the respondent's lifetime that the respondent would be willing to forego to live in a perfect health state and avoid living in a bad health state. The respondent is asked to choose between 2 health states (ie, a perfect health state [choice A] and an intermediate health state [choice B]) (Fig. 2). The TTO is an iterative process where the intermediate health state (choice B) always remains the same (ie, only the life span varies). The first step is to compare the 2 health states with the same lifetime (ie, the maximum duration of 10 years). The lifetime corresponding to the perfect health state will then be decreased or increased according to the answers given. For the second TTO question, if the respondent indicates that he/she prefers the perfect health state with no life expectancy (ie, immediate death) to the proposed intermediate health state, he/she will be offered a new format for the TTO with the same intermediate health state. In this new format, the maximum lifespan is extended to 20 years and the respondent has to choose between the perfect health state (choice A) and a combination of the perfect health state and the intermediate health state (ie, 10 years in the perfect health state followed by 10 years in the intermediate health state) (choice B) (Fig. 3). The lifespan in the perfect health state will then vary according to the individual's responses.

**Figure 4:**
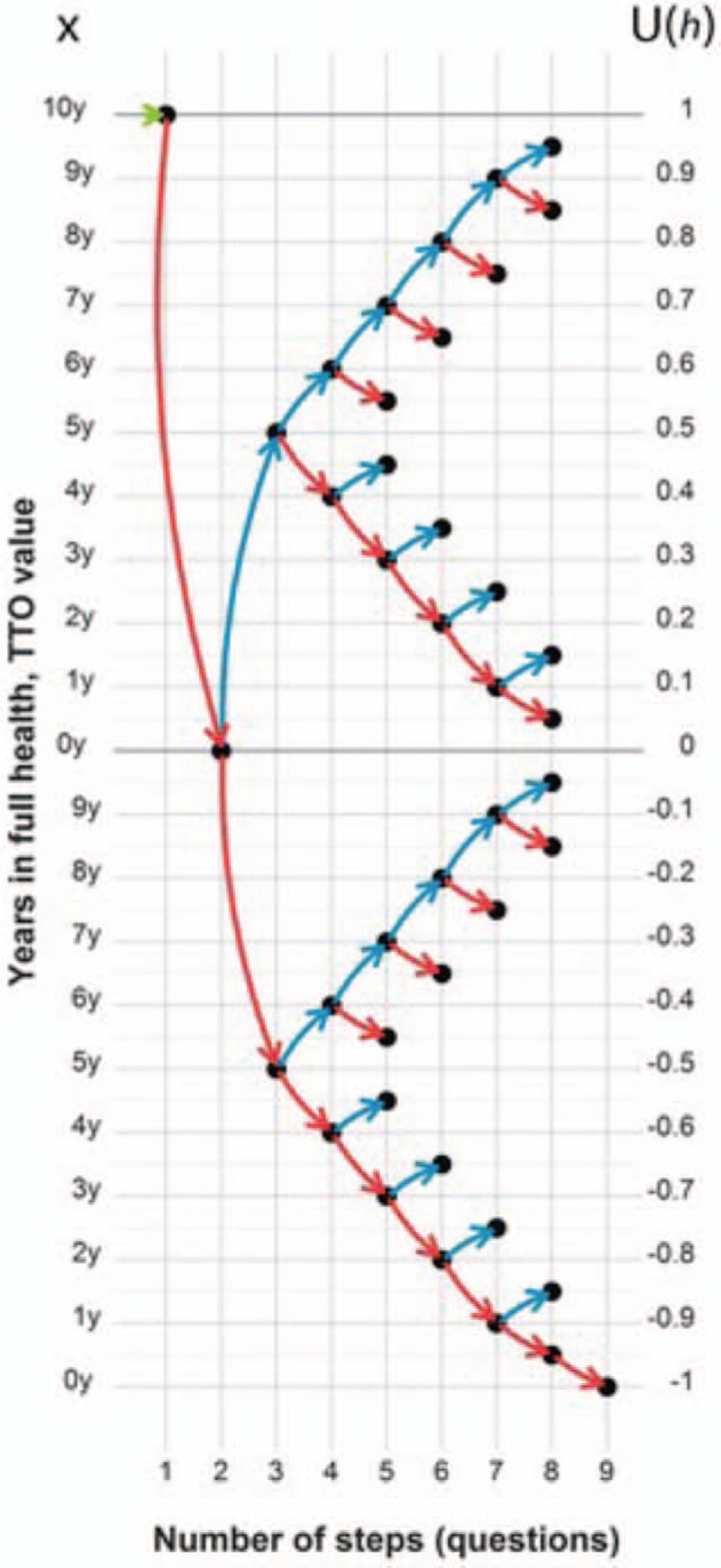
The sequence of choice using the time-trade-off (TTO) choice method. Red arrows represent choice A and blue ones choice B. The figure was adapted from Oppe et al.^[40]^

This procedure will be repeated for each health state that one wishes to evaluate, and also for the worst possible health state. In total, each respondent will have 9 TTO procedures to perform (ie, 7 intermediate health states, the current health status of the patient as assessed by the SF-6Dv2, and the worst possible health state). The iterative process of respondent choices is described in Fig. 4, adapted from the study by Oppe et al.^[40]^ Red arrows represent choice A (ie, the perfect health state) and blue ones choice B (ie, the intermediate health state). The possible utility values range from −1 to 1 in 0.05 increments.

#### 2.5.5 The DCE method

**Figure 5:**
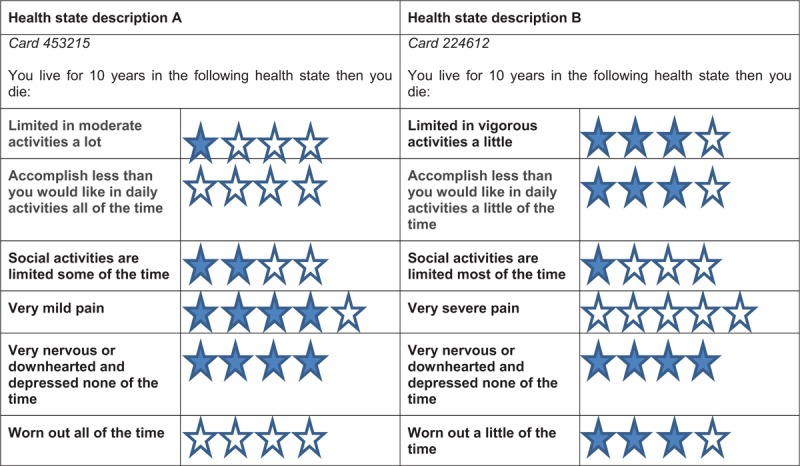
Health state cards choice using the discrete choice experiment (DCE) method.

The version of the DCE is a simple version of the one used by Norman et al.^[41]^ In this study, each respondent will be presented with two intermediate health states described by the SF-6Dv2 classification (Fig. 5). The life span in these health states will always be 10 years. Each respondent will then be asked to choose between these 2 intermediate health states (ie, the one in which they would prefer to live). This step will be repeated 7 times with different pairs of intermediate health states.

The TTO and DCE results will be combined via a hybrid approach, using the method developed by Ramos-Goñi et al.^[42]^ This method will integrate the interval responses from the TTO with the dichotomous responses from the DCE under a common likelihood specification. The hybrid model will maximize a single likelihood function. The model also allows the dichotomous and continuous responses to have different distributions (logistic and normal) and have different independent variables to model the scaling terms.

#### 2.5.6 Selection of health state cards and sample size

The different intermediate health states used in this study will come from the 18,750 possibilities offered by the choice of answers to the SF-6Dv2 questionnaire (5 statements with 5 levels and 1 statement with 6 levels: 5^5^ × 6). Because it is not possible to evaluate all of the 18,750 possible health states or the 351.5 million (18,750 × 18,749) sets of possible pairs for the DCE, the selected health states and pairs are the results of an orthogonal selection procedure. This procedure allows the identification of a model to estimate the value of all the health states defined by the SF-6Dv2 (ie, an orthogonal main effects design). To perform an efficient estimation, the number of pairs to be evaluated must be greater than the number of parameters to be estimated for the DCE. Because we will use 6 health dimensions with 5 or 6 levels each, the minimum number of parameters to be estimated is 26 (ie, 1 + sum of the total effect of dimensions, 5 × [5 − 1] + [6–1] = 25). For the TTO, there is no minimum number of health conditions to be assessed; however, this is much more dependent on the minimum number of health states suggested during the orthogonal selection procedure, and, in the literature, a minimum of 49 is often noted,^[32]^ with generally about 200 to 300 evaluated health states.^[28,43]^ To comply with these minimum figures, we performed an orthogonal selection procedure that yielded 216 intermediate health states with a D-efficiency of 99.93%. From these intermediate health states, the orthogonal selection procedure allowed us to design 70 pairs for the DCE, thus leaving 76 health states for the TTO procedures. These figures are beyond the minimum required and will allow us to randomly select 7 intermediate health states from the 76 in each TTO set and 10 pairs of intermediate health states from the 70 in each DCE set. By doing so, each respondent will evaluate 21 intermediate health states plus his/her own health state and the worst possible health state.

The optimal sample size is more difficult to calculate here because it is not a question of carrying out a simple power test with a pre-established hypothesis, but of ensuring the efficiency of the estimates. Lancsar and Louvière ^[44]^ suggest that a number of 20 answers per choice card is sufficient in a DCE to make efficient estimates, whereas Burgess et al^[45]^ indicate that 10 would be sufficient. Considering these indications, a minimum of 15 respondents per choice card is retained in this study. Consequently, a minimum of 105 respondents will be necessary for the DCE in each group of cancer patients (ie, 70 pairs × 15 respondents/number of random blocks = 70 × 15/10 = 105). The same number of respondents will be required for the TTO.

### 2.6 Data analysis

The collected data will be compiled and filtered in Excel and transferred to the StataSE14 statistical analysis software for estimation and efficiency testing (StataCorp, TX). The preferred estimation model will be the one developed by Ramos-Goñi et al.^[42]^ Various methods, including those used by Brazier et al^[28]^ and Norman et al,^[41]^ will be tested using a random-effects model with or without the interactive term MOST^[28]^ and probit models.^[41]^ The parsimonious model will also be tested.^[29]^ As an indication of the efficiency of estimates, we will calculate the mean absolute error (MAE) and count the number of absolute errors greater than 0.05 and 0.10. The consistency of the results will be assessed by identifying whether the size of the coefficient is growing while the description of the health state moves toward perfect health; the number of inconsistencies will be reported.

To determine the existence of differences in cancer health utilities between groups, subgroup analyses will be performed by multiple regression analyses, covariance analyses, and comparison of the coefficients associated with the different SF-6Dv2 levels when estimates are made in the subgroups.

For the analysis of the SF-6Dv2-associated utilities in relation to the other HRQoL questionnaires, Pearson correlation tests will be performed. Generalized estimating equations (GEE) will also be used to take into account the cluster effect in questionnaire scores for the same person. Bland-Altman graphs will be produced to describe the differences between different HRQoL questionnaires. Also, to carry out the mapping of HRQoL-specific questionnaires associated with their modules (ie, the EORTC QLQ-C30 and FACT-G) with the SF-6Dv2, different regressions will be performed to evaluate the predictive effect of the SF-6Dv2 utilities on the scores obtained for the different dimensions estimated by these specific questionnaires. The model performance will be measured by evaluating the predictive capacity using root mean square error and the MAE and by analyzing the explanatory power via the adjusted *R*^2^ values. Estimates will be computed with ordinary least squares methods using an additive model and the addition of multiplicative variables will be tested. The best models will be retained so that a QALY calculation can be derived from these specific HRQoL questionnaires in the Quebec context.

### 2.7 Ethical and dissemination

The proposed research was reviewed and approved by the Institutional Research Ethics Review Board of our institution. Research participants will be duly informed and written consent will be obtained before the survey. The director of professional services in our institution authorized a prescreening of patients and access to appointment lists to allow us to determine each potentially eligible patient. The survey completion will be done using a personalized code, and no patient name or medical identifier will appear in the database. The key associating the personalized code and sensitive data of patients will be collected in a secure Excel file separately from the database and only the people doing the recruiting will have access to it. All collected paper information will be kept in a locked room. When analyzing the results, respondents will be listed as a group.

### 2.8 Ethics approval and consent to participate

This study has been reviewed and approved by the Institutional Research Ethics Review Board of the CHUS (2017–1490). All participants will sign a consent form. All participants to recruit will provide their consent for publication.

## 3 Discussion

The main objective of this study is to develop a preference weights dataset for QALY congruent with the Quebec linguistic and sociocultural context of patients with cancer and to compare it with a general population dataset (forthcoming study with a similar survey). The development of such a dataset will allow physicians and decision-makers to conduct CUA using utility values adapted to the context of patients with cancer in Quebec, which will, in turn, allow physicians and decision-makers to choose between the different possible interventions based on the real preferences of the individuals concerned.

However, this study has some limitations. There may be internal validity bias in considering a risk of a “scope effect” problem for some respondents if they misinterpret certain questions. To avoid this problem, the choice cards will be conscientiously explained during the first round of the survey and help will be provided to complete the first choice tasks. Also, research assistants will explain to respondents the meanings of the different symbols used. Furthermore, to control this bias, we will ask at the end of the survey if the respondent had difficulty answering and if he/she felt irritated or bored by the process. Respondents who indicate that the exercise is too difficult will be excluded from the analysis.

There could also be an external validity bias. Indeed, some subjects could refuse to participate for a variety of personal reasons, and there is a risk that some questionnaires may not be fully completed or in sufficient number. Finally, some respondents may decide to respond quickly to the questionnaire by always selecting the same choices. To avoid this bias, all identical answer sets will be excluded from the analysis.

## 4 Conclusions

This study will be the first to allow researchers to develop a preference weights dataset for a QALY instrument in the context of cancer patients in Quebec. This instrument will be better adapted than existing ones because it will allow practitioners to consider the needs and preferences of Quebecers suffering from cancer in the measure of their HRQoL while meeting the requirements of economic theory on resource allocation in the context of competition between different care alternatives and budget constraints. Furthermore, this will be very useful for decision-makers and medical doctors, because it is now advocated to consider both the general population and patient preferences in CUAs.^[21]^ Finally, this start-up work may also initiate similar projects on other pathologies.

## Acknowledgments

We would like to thank all those who contributed to this study.

## Author contributions

TGP is member of the FRQS-funded Centre de recherche du CHUS. TGP conceived the study. All authors contributed to its design and approved the final manuscript.

**Conceptualization:** Thomas G Poder, Nathalie McFadden, Michel Pavic.

**Data curation:** Thomas G Poder.

**Formal analysis:** Thomas G Poder.

**Funding acquisition:** Thomas G Poder, Nathalie McFadden, Michel Pavic.

**Investigation:** Thomas G Poder.

**Methodology:** Thomas G Poder, Nathalie Carrier.

**Project administration:** Thomas G Poder, Nathalie Carrier, Nathalie McFadden, Michel Pavic.

**Resources:** Thomas G Poder.

**Software:** Thomas G Poder.

**Supervision:** Thomas G Poder.

**Validation:** Thomas G Poder, Michel Pavic.

**Visualization:** Thomas G Poder.

**Writing – original draft:** Thomas G Poder, Nathalie Carrier.

**Writing – review & editing:** Thomas G Poder, Nathalie Carrier, Nathalie McFadden, Michel Pavic.

## References

[1] Brazier J Ratcliffe J Saloman J Measuring and Valuing Health Benefits for Economic Evaluation. Second Edition. Oxford University Press: New York; 2007.

[2] McHorney CA Ware JE Lu JF The MOS 36-item Short-Form Health Survey (SF-36): III. Tests of data quality, scaling assumptions, and reliability across diverse patient groups. Med Care 1994;32:40–66. 8277801 10.1097/00005650-199401000-00004

[3] Aaronson NK Ahmedzai S Bergman B The European Organization for Research and Treatment of Cancer QLQ-C30: a quality-of-life instrument for use in international clinical trials in oncology. J Natl Cancer Inst 1993;85:365–76. 8433390 10.1093/jnci/85.5.365

[4] Osoba D Aaronson N Zee B Modification of the EORTC QLQ-C30 (version 2.0) based on content validity and reliability testing in large samples of patients with cancer. The Study Group on Quality of Life of the EORTC and the Symptom Control and Quality of Life Committees of the NCI of Canada Clinical Trials Group. Qual Life Res 1997;6:103–8. 9161109 10.1023/a:1026429831234

[5] Cella DF Tulsky DS Gray G The Functional Assessment of Cancer Therapy scale: development and validation of the general measure. J Clin Oncol 1993;11:570–9. 8445433 10.1200/JCO.1993.11.3.570

[6] Conroy T Mercier M Bonneterre J French version of FACT-G: validation and comparison with other cancer-specific instruments. Eur J Cancer 2004;40:2243–52. 15454249 10.1016/j.ejca.2004.06.010

[7] Schipper H Clinch J McMurray A Measuring the quality of life of cancer patients: the Functional Living Index-Cancer: development and validation. J Clin Oncol 1984;2:472–83. 6374052 10.1200/JCO.1984.2.5.472

[8] Mercier M Bonneterre J Schraub S The development of a French version of a questionnaire on the quality of life in cancerology (Functional Living Index-Cancer: FLIC). Bull Cancer 1998;85:180–6. 9752337

[9] Brazier J The Short-Form 36 (SF-36) Health Survey and its use in pharmacoeconomic evaluation. Pharmacoeconomics 1995;7:403–15. 10155328 10.2165/00019053-199507050-00005

[10] Spilker B Quality of Life and Pharmacoeconomics in Clinical Trials. Philadelphia: Lippincott-Raven Publishers; 1996.

[11] Fayers P Hays R Assessing Quality of Life in Clinical Trials. 2nd ed. New York: Oxford Press; 2005.

[12] Ware J Kinsinski M Bjorner J User's manual for the SF-36v2 Health Survey. 2nd ed. Lincoln: RI: QualityMetric Incorporated; 2007.

[13] Tolley K What are Health Utilities? London: Hayward Medical Communications; 2009.

[14] Herdman M Gudex C Lloyd A Development and preliminary testing of the new five-level version of EQ-5D (EQ-5D-5L). Qual Life Res 2011;20:1727–36. 21479777 10.1007/s11136-011-9903-x PMC3220807

[15] Zarate V Kind P Chuang L-H Hispanic valuation of the EQ-5D health states: a social value set for Latin Americans. Value Health 2008;11:1170–7. 18489516 10.1111/j.1524-4733.2008.00349.x

[16] Glied S Smith PC The Oxford Handbook of Health Economics. New York: Oxford University Press; 2013.

[17] Torrance GW Measurement of health state utilities for economic appraisal. J Health Econ 1986;5:1–30. 10311607 10.1016/0167-6296(86)90020-2

[18] Drummond MF Sculpher MJ Torrance G Methods for the Economic Evaluation of Health care Programmes. 3rd ed. New York: Oxford University Press; 2005.

[19] Muennig P Cost-effectiveness Analyses in Health: a Practical Approach. 2nd ed. San Francisco: Jossey Bass; 2008.

[20] Drummond M Brixner D Gold M Toward a consensus on the QALY. Value Health 2009;12 Suppl 1:S31–35. 10.1111/j.1524-4733.2009.00522.x 19250129

[21] Versteegh MM Brouwer WBF Patient and general public preferences for health states: a call to reconsider current guidelines. Soc Sci Med 2016;165:66–74. 27497260 10.1016/j.socscimed.2016.07.043

[22] Rowen D Mulhern B Banerjee S Comparison of general population, patient, and carer utility values for dementia health states. Med Decis Making 2015;35:68–80. 25385749 10.1177/0272989X14557178 PMC4270996

[23] Stein JD Brown MM Brown GC Quality of life with macular degeneration: perceptions of patients, clinicians, and community members. Br J Ophthalmol 2003;87:8–12. 12488253 10.1136/bjo.87.1.8 PMC1771467

[24] Garau M Shah KK Mason AR Using QALYs in cancer: a review of the methodological limitations. Pharmacoeconomics 2011;29:673–85. 21599035 10.2165/11588250-000000000-00000

[25] Aburub AS Gagnon B Rodríguez AM Using a personalized measure (Patient Generated Index (PGI)) to identify what matters to people with cancer. Support Care Cancer 2016;24:437–45. 26099901 10.1007/s00520-015-2821-7

[26] Butt T Dunbar HMP Morris S Patient and public preferences for health states associated with AMD. Optom Vis Sci 2013;90:855–60. 23811607 10.1097/OPX.0b013e3182962318

[27] Brazier J Usherwood T Harper R Deriving a preference-based single index from the UK SF-36 Health Survey. J Clin Epidemiol 1998;51:1115–28. 9817129 10.1016/s0895-4356(98)00103-6

[28] Brazier J Roberts J Deverill M The estimation of a preference-based measure of health from the SF-36. J Health Econ 2002;21:271–92. 11939242 10.1016/s0167-6296(01)00130-8

[29] Brazier JE Roberts J The estimation of a preference-based measure of health from the SF-12. Med Care 2004;42:851–9. 15319610 10.1097/01.mlr.0000135827.18610.0d

[30] Poder TG Gandji EW PHP145-SF6D value sets: a systematic review. Value Health 2016;19:A282.

[31] Ferreira LN Ferreira PL Pereira LN A Portuguese value set for the SF-6D. Value Health 2010;13:624–30. 20230545 10.1111/j.1524-4733.2010.00701.x

[32] Lam CLK Brazier J McGhee SM Valuation of the SF-6D health states is feasible, acceptable, reliable, and valid in a Chinese population. Value Health 2008;11:295–303. 18380642 10.1111/j.1524-4733.2007.00233.x

[33] Abellán Perpiñán JM Sánchez Martínez FI Martínez Pérez JE Lowering the “floor” of the SF-6D scoring algorithm using a lottery equivalent method. Health Econ 2012;21:1271–85. 21976290 10.1002/hec.1792

[34] Holzner B Kemmler G Sperner-Unterweger B Quality of life measurement in oncology: a matter of the assessment instrument? Eur J Cancer 2001;37:2349–56. 11720827 10.1016/s0959-8049(01)00307-0

[35] Questionnaires: Cancer Specific Measures. FACIT.org. Available at: http://www.facit.org/facitorg/questionnaires.

[36] EORTC QLQ-C30. EORTC Quality of Life. Available at: http://groups.eortc.be/qol/eortc-qlq-c30.

[37] Mulhern B Brazier J Developing version 2 of the SF-6D: the health state classification system. Qual Life Res 2014;23:49.23912852

[38] Bansback N Mulhern B Sawatsky R Valuing the SF-6Dv2 in Canada. Qual Life Res 2015;24:181–91.25048731

[39] Luo N Ramos-Goni J Some new strategies for eliciting and modeling utility values of multi-attribute health states 2016. Presented at the ISPOR 21st International Meeting Program. Workshop session V, W31.

[40] Oppe M Rand-Hendriksen K Shah K EuroQol protocols for time trade-off valuation of health outcomes. Pharmacoeconomics 2016;34:993–1004. 27084198 10.1007/s40273-016-0404-1 PMC5023738

[41] Norman R Viney R Brazier J Valuing SF-6D health states using a discrete choice experiment. Med Decis Making 2014;34:773–86. 24025661 10.1177/0272989X13503499

[42] Ramos-Goñi JM Craig B Oppe M Combining continuous and dichotomous responses in a hybrid model. EuroQol Res Foundation 2016.

[43] Wong ELY Ramos-Goñi JM Cheung AWL Assessing the use of a feedback module to model EQ-5D-5L health states values in Hong Kong. Patient 2018;11:235–47. 29019161 10.1007/s40271-017-0278-0 PMC5845074

[44] Lancsar E Louviere J Conducting discrete choice experiments to inform healthcare decision making: a user's guide. Pharmacoeconomics 2008;26:661–77. 18620460 10.2165/00019053-200826080-00004

[45] Burgess L Street DJ Wasi N Comparing designs for choice experiments: a case study. J Stat Theory Pract 2011;5:25–46.

